# The development of a visual dashboard report to assess physician assistant and nurse practitioner financial and clinical productivity

**DOI:** 10.1186/s12913-022-08216-7

**Published:** 2022-07-08

**Authors:** Vasco Deon Kidd, Joe Haoming Liu, Andy Reamer-Yu, Joann Hao Wang, Mei Deng

**Affiliations:** 1 School of Medicine, Department of Orthopaedic Surgery, Office of Advanced Practice, University of California Irvine (UCI Health), 101 The City Dr S, Orange, CA 92868 USA; 2 Decision Support Services, Dean’s Office, School of Medicine, University of California Irvine (UCI Health), 101 The City Dr S, Orange, CA 92868 USA; 3 Decision Support Services, Dean’s Office, School of Medicine, University of California Irvine (UCI Health), 101 The City Dr S, Orange, CA 92868 USA; 4 Office of Advanced Practice, University of California Irvine (UCI Health), 101 The City Dr S, Orange, CA 92868 USA

**Keywords:** Physician assistant, Physician associate, Nurse practitioner, Advanced practice provider, Dashboard, Visualization analytics, Relative value unit, Academic medical center

## Abstract

The evolving COVID-19 pandemic has unevenly affected academic medical centers (AMCs), which are experiencing resource-constraints and liquidity challenges while at the same time facing high pressures to improve patient access and clinical outcomes. Technological advancements in the field of data analytics can enable AMCs to achieve operational efficiencies and improve bottom-line expectations. While there are vetted analytical tools available to track physician productivity, there is a significant paucity of analytical instruments described in the literature to adequately track clinical and financial productivity of physician assistants (PAs) and nurse practitioners (NPs) employed at AMCs. Moreover, there is no general guidance on the development of a dashboard to track PA/NP clinical and financial productivity at the individual, department, or enterprise level. At our institution, there was insufficient tracking of PA/NP productivity across many clinical areas within the enterprise. Thus, the aim of the project is to leverage our institution’s existing visualization tools coupled with the right analytics to track PA/NP productivity trends using a dashboard report.

**Methods**

We created an intuitive and customizable highly visual clinical/financial analytical dashboard to track productivity of PAs/NPs employed at our AMC.

**Results**

The APP financial and clinical dashboard is organized into two main components. The volume-based key performance indicators (KPIs) included work relative value units (wRVUs), gross charges, collections (payments), and payer-mix. The session utilization (KPIs) included (e.g., new versus return patient ratios, encounter type, visit volume, and visits per session by provider). After successful piloting, the dashboard was deployed across multiple specialty areas and results showed improved data transparency and reliable tracking of PAs/NPs productivity across the enterprise. The dashboard analytics were also helpful in assessing PA/NP recruitment requests, independent practice sessions, and performance expectations.

**Conclusion**

To our knowledge, this is the first paper to highlight steps AMCs can take in developing, validating, and deploying a financial/clinical dashboard specific to PAs/NPs. However, empirical research is needed to assess the impact of qualitative and quantitative dashboards on provider engagement, revenue, and quality of care.

## Background

Physician assistants (PAs) and nurse practitioners (NPs) often referred to as advanced practice providers (or APPs) have been a part of organized medicine in the United States since the late 1960’s. Nurse practitioners are advanced practice registered nurses (APRNs) who are graduate-educated in a specific population foci (e.g., pediatrics, acute care, women's health, family), whereas PAs are graduate-educated as a “generalist” in the medical model, which complements physician training [1]. These providers are an integral part of a team-based approach and are employed in almost all medical and surgical settings within the United States [2]. Health care services provided by PAs/NPs are reimbursed by insurers at rates slightly lower than that of a physician. Research consistently demonstrates that patients receiving care from PAs/NPs have high satisfaction rates and clinical outcomes comparable to that of primary care physicians [3–5]. Although, AMCs have employed PAs/NPs for decades, the vast majority of AMCs have not successfully documented the financial impact or outcomes associated with individual PA/NP care [6]. In today's evolving healthcare environment, AMCs nationwide are taking cost-cutting measures to try to recoup lost revenue from canceled surgeries and higher care costs for treating patients with more severe conditions [7]. Given the year-over-year declines in provider productivity experienced by some hospital systems because of the pandemic, it’s imperative AMCs enhance KPI tracking and consistency to identify opportunities to improve profitability, population health, clinical outcomes, patient access and experience. A failure to appreciate, which KPI metrics drive improvement efforts and effect real change can lead to undesirable organizational outcomes such as insufficient clinical productivity and revenue growth.

In the United States, the relative value unit (RVU) is the most popular payment model for most types of clinical practice activities as each Current Procedural Terminology (CPT) code is assigned an RVU, which is multiplied by a conversion factor and geographic adjustment to determine the Medicare-allowed payment [8]. RVUs are often used in compensation formulas and productivity metrics by AMCs, however, there is a growing recognition of the potential impacts of a wRVU-based compensation model on unremunerated activities, such as teaching and scholarship [9]. Unlike faculty physicians, most APPs employed at AMCs do not receive protected time for academic activities and are expected to carry out the clinical mission of the enterprise.

The aim of this article is to discuss the development of a highly visual APP dashboard report coupled with financial KPI metrics to gain a more transparent view into APP financial productivity and potentially drive practice improvements at our institution. Prior to this engagement, our institution consistently struggled to track and report APP clinical effort and the impact to productivity and revenue. Moreover, APPs requested more feedback regarding their productivity.

### Methodology

In collaboration with the office of advanced practice and decision support services (DSS) team, we created an electronic interactive financial dashboard to track financial and clinical utilization data for 140 APPs at our institution. The financial dashboard underwent pilot testing and internal data validation before enterprise rollout. The new dashboard provided the following information: gross charges, payments (collections), wRVUs, payer-mix, and session statistics.

### DSS home-growth system

The *School of Medicine – Decision Support Services* is an internal website that provides web tools, reports, and dashboards to support the College of Health Science’s and School of Medicine’s goals and decision-making processes. The web tools are used for budgeting, recruitments, and employee related activities. The reports and dashboards provide analysis and analytics to support decision making.

### Dashboard design

**Fig. 1:**
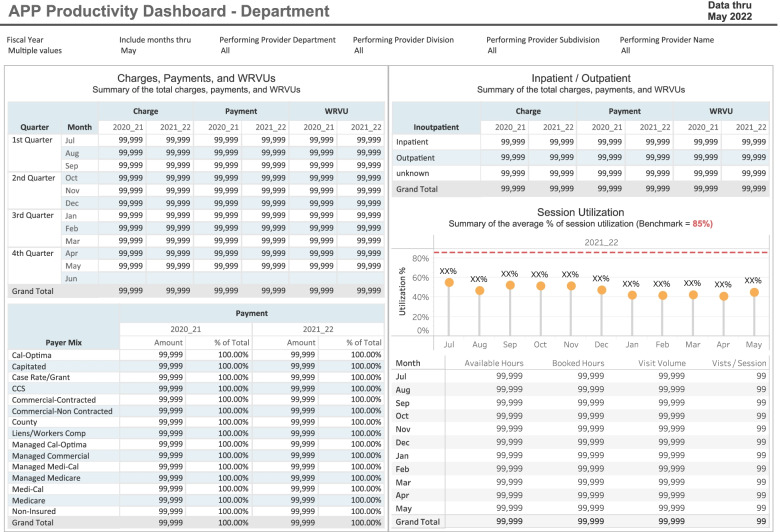
The APP productivity dashboard demonstrating multiple volume-based key performance indicators (KPI) and session statistics

**Fig. 2:**
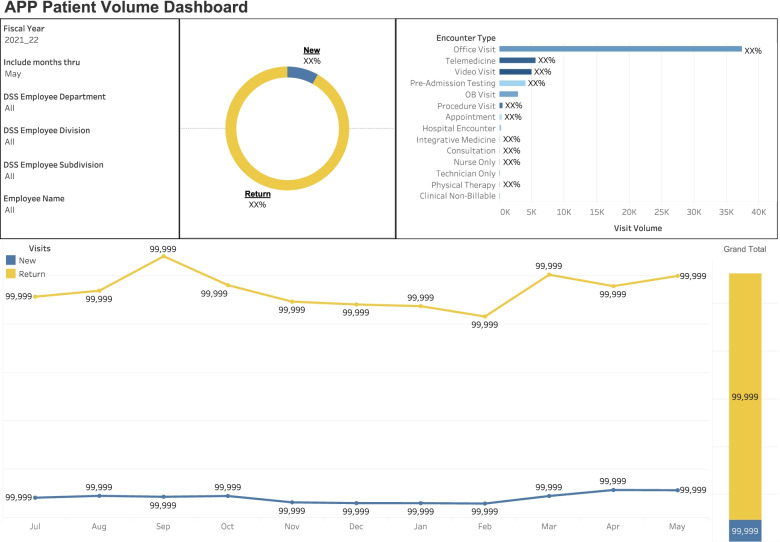
The APP patient volume dashboard demonstrating new patient vs established patient visits and encounter type metrics

Multiple stakeholders were involved in either the design, review, and/or pilot phase of the dashboard project. The stakeholders included clinically practicing PAs and NPs, decision support team, director of advanced practice, financial analyst from office of advanced practice, and Health Affairs Information Systems (HAIS -Team). After several iterations through the prototyping dashboard design process, we developed an interactive dashboard report that can track monthly and daily focused KPIs at the individual APP, department, and/or enterprise level. The session utilization (KPIs) included (e.g. *new versus return patient ratios, encounter type, booked/available hours, visit volume, and visits per session by provider)*. The volume-based key performance indicators (KPI) included (*e.g., wRVU’s, gross charges, collections (payments), and payer mix).* The Payor mix classifications include (medicare, medi-cal, commercial-non, contracted, commercial contracted, noninsured, etc.). The dashboard mainly tracks ambulatory metrics and some inpatient data reporting. The interactive dashboard allows APPs to apply filtering functions quickly and easily to evaluate multiple content areas and metrics concurrently but does not measure care experience directly. The dashboard interface is characterized by straightforward graphics. The dashboard does not include a peer-to-peer data comparison option for end users. We have included a visual representation of the active APP dashboard (Figs. 1 and 2).

All financial data is sourced from PBG data mart after month-end close. Clinical data is sourced directly from Epic (our Health’s EHR vendor) data mart daily. Epic is used by more than 250 health care organizations nationwide. Additionally, the director of advanced practice providers oversees all updates to the dashboard report including, aesthetic data labels, displaying of data, and ensuring the content aligns with the purpose of dashboard.

### APP dashboard process flow

The source data of the APP dashboard is a combination of clinical billing data from School of Medicine (SOM), physician billing group (PBG), non-clinical billing data from EPIC, and employee data from our payroll and personnel system. The source data is loaded into the SOM DSS database via extract-transform-load (ETL) procedures.

Once data is loaded into the SOM DSS database, a query is used to combine the various data source for the APP dashboard. Once this is ready, Tableau (SOM’s vendor for reporting and analytics) is used to create the dashboard and is published to the SOM DSS website for the individual APP to view and access their metrics.

#### Pulling data (professional billing) by performing provider versus billing provider

SOM DSS team had rebuilt an internal data mart containing provider/faculty clinical professional billing data in the SOM DSS internal warehouse. In October 2020, it was decided to use the current clinical professional billing data mart and retrieved data set, which is pulled by performing provider since our APPs are listed as the “performing provider” and have weekly independent clinic sessions. In healthcare, the performing provider (rendering provider) is classified as an independently licensed practitioner either a physician or APP who personally performs the health care service directly to a patient. The performing provider classification ensures that APP productivity is captured even though some payers’ billing policies require the APP bill under the credentialed supervising physician’s national provider identifier (NPI), which renders the APP’s work contribution invisible in the claims data. Additionally, APPs participating in split/shared billing (evaluation and management (E/M) services performed jointly between a physician and APP) are not currently captured within the dashboard as it is difficult to tease out the contribution of the APP in this model. However, under the new The Centers for Medicare & Medicaid Services (CMS) changes in 2022, a Split Shared Modifier FS is now a requirement with claims for split shared services performed in facility settings. The new modifier will provide greater transparency in tracking of physician/APP time spent on a visit.

#### Pulling data for session utilization data workstream

**Fig. 3:**
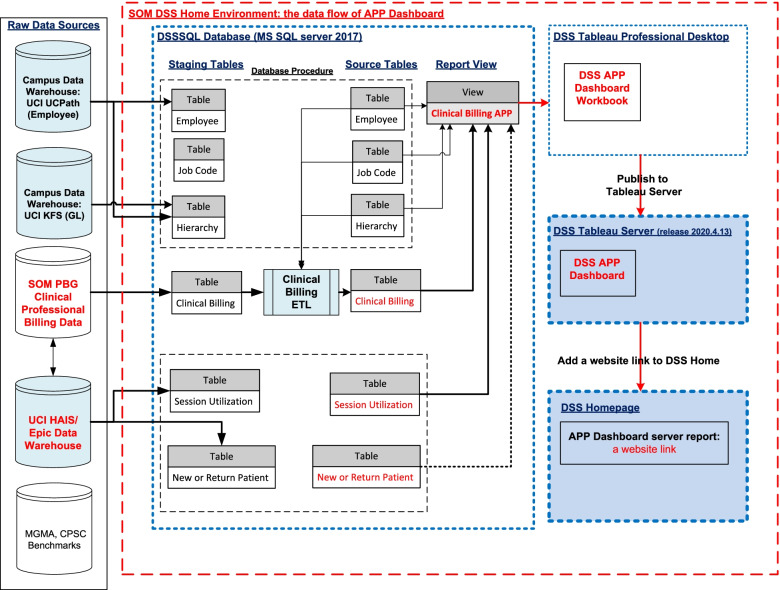
The data flow to the APP dashboard

In Q1 and Q2 of 2021, the SOM DSS team worked with Medical Center’s Clinical Informatics and Enterprise Data and Analytics team to retrieve session utilization data. Session utilization data is sourced from our Epic system (Fig. 3).

### Data Usability

Cohorting data by school of medicine department or division is a dashboard feature.

- Based on the home department of each APP as performing provider in our clinical data mart.

Aggregating data across all providers in all service locations.

- Data is based on PBG’s month-end clinical professional billing data into the SOM DSS clinical data mart.

Monthly capture of dashboard data and potential lag times.

- Session utilization by 8am daily after Epic data mart nightly reloading.
- New and return patients data by 8am daily after Epic data mart nightly reloading.
- Home department assignment of APPs are retrieved from employee data in our campus data warehouse weekly.
- Organization hierarchy are from the Kuali Finance System (KFS) Financial system daily and campus data warehouse weekly.

### Data validation and Integrity

The SOM DSS team has validations in place to ensure the integrity of the data. The data validation process begins when the clinical billing data is sent from PBG to the DSS team where a reconciliation process is done between both sides. PBG provides the number of records and amounts related to the financial data that was sent and the DSS team will compare the information with what was loaded into their system. If any discrepancies exist, both teams will work on verifying the cause of the data mismatch. Other types of validation include, verifying the results with other existing dashboards that are made available at the institution to ensure that the resulting outcome matches with the data found on the APP dashboard. Additionally, Ernst and Young consultants also validated our clinical billing data between PBG and DSS systems in the past 2 to 3 years, and works closely with, Dean’s Office Finance and Clinical Affairs team. Furthermore, six weeks after deployment of the dashboard, the analyst from the office of advanced practice along with a consultant from The Chartis Group, a leading healthcare advisory firm validated dashboard financial data (charges, payments, and wRVUs) across three selected clinical specialties.

In addition, the clinic session data was added to the SOM DSS data mart and tested between March 2021 and August 2021. The new and return patients data was added to the SOM DSS data mart and tested in 2021 Q3 and Q4. These embedded check and balances ensure the data is accurate and reliable.

### Differences and similarities between organizational dashboards

The dashboard used by both the physician and APP have similarities in that both show financial clinical billing data (e.g., charges, payments, wRVUs) that allows clinicians to view trends over time. Unlike the APP dashboard, the physician dashboard does not include session statistics such as volume data broken down by new or return visits and encounter types (e.g., office visits, telemedicine, procedure visit, etc.) but does include CPT code details not available on the APP dashboard.

### APP dashboard testing and system-wide roll-out

We had a successful product launch of the APP dashboard system-wide. Instructions for accessing the dashboard were embedded within our quarterly advanced practice newsletter. During the open comment period, there were no issues accessing the dashboard by SOM employed APPs. However, APPs practicing within the federal qualified Health Center (FQHC) were unable to access their dashboard metrics as the FQHC's clinical billing data is not captured through PBG. In addition, APPs exclusively employed to conduct preoperative chart reviews where no clinical pro-fee billing exists, have no data displayed in their dashboards.

### Privacy and security

**Fig. 4:**
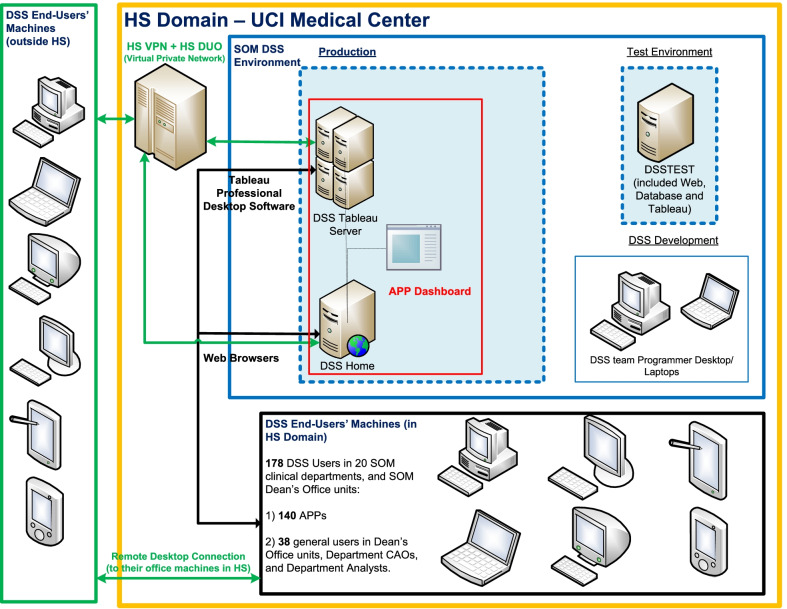
The privacy and security features to access the APP dashboard

The dashboard is available to the individual APP via a secured employee electronic identification (ID) in the result set and protected from unauthorized access. When an APP views their dashboard, a verification is done to match their user ID with the one in the result set. If the user ID matches, then all data related to that user ID is displayed, otherwise nothing will be shown in the dashboard. The dashboard is behind a virtual private network (VPN), and requires an organization issued computer to access the VPN. If an APP is outside of the VPN, they will not be able to access their dashboard. The VPN has a two factor authentication set-up using the software from Duo Security https://duo.com/product/multi-factor-authentication-mfa/two-factor-authentication-2fa. The duo security feature uses a specific type of multifactor authentication (MFA) (Fig. 4).

## Discussion

To our knowledge, this is the first article to discuss the development, and deployment of an APP specific dashboard at an AMC. The comprehensive dashboard enables APPs and administrators to access and compare APP productivity metrics against department standard performance expectations without having to extract data from multiple sources to compute wRVUs. Dashboard acceptance and usage has increased significantly among our outpatient APPs and managers since the initial go-live in 2021. The dashboard is particularly helpful in providing a consolidated view of financial and clinical data, which aligns with the needs of end users. One immediate benefit to end users occurred because of an institutional change to align the APP funds flow model with top-of-license practice for outpatient APPs, which began shortly after the implementation of the dashboard.

To help offset department-paid APP salary/benefit expenses, departments are now paid a dollar per wRVU ($/wRVU) payment based on the wRVUs generated by the outpatient APP as the performing provider, and at the appropriate $/wRVU benchmark (general or specialty rate). The department also receives 13% of collections associated with the outpatient APPs revenue by billing provider to offset other departmental expenses. Tracking total APP wRVUs and net payments is essential in calculating breakeven point. Needless to say, non-RVU generating activities are not captured within the dashboard. The APP dashboard allows end users to review comparison data for a selected time period. For example, at the enterprise level, APP productivity year-over-year aggregate data across 6 departments demonstrated a 115% increase in wRVUs, 77% increase in payments, and 100% increase in charges from the prior fiscal year (July to February). It should also be noted that the increase in APP productivity did not negatively impact the physician productivity model. In light of the new funding changes, APPs have found it easier to access and monitor their KPI metrics through the dashboard.

Another benefit of our dashboard is the ability to track the required number of APP independent clinic sessions required under our newly adopted ambulatory practice standards. Under the new guidelines, a clinical full-time equivalent (cFTE) ambulatory APP is expected to have nine independent clinic sessions totaling 36 patient contact hours each week. This equates to a monthly total of 144 available hours on an APP’s template to schedule patients. In addition to available hours, the dashboard tracks the number of booked hours defined as the total number of hours that the APP is scheduled to see patients. These dashboard metrics are extremely useful in identifying departmental adherence to and compliance with the new enterprise practice standards, and pinpointing opportunities for redistributing resources, improving session standardization, and template optimization.

The dashboard report along with other factors is also used as a resource in evaluating recruitment requests for net new or replacement APP career and non-career positions. The dashboard’s template utilization percentage (booked hours divided by available hours) with an anticipated goal of 85% template utilization is particularly helpful in identifying and assessing untapped capacity that may exist within a department or division to absorb incremental increases in patient volume, which may negate or necessitate the need to hire an additional APP.

Lastly, while lag times are prevalent within dashboard reporting systems, our institutional data lag time (4 weeks) did not negatively impact dashboard performance or acceptance.

### Limitations

While we acknowledge the generalizability of our article may be limited due to our institution’s experience with creating an APP dashboard, this report can serve as a guide to other healthcare organizations eager to monitor and measure APP productivity through the development of a customizable dashboard report. Additionally, the paper serves as a springboard for further investigation and research on this topic.

## Conclusions

Given the double-digit growth of APPs in the healthcare workforce over the last decade, it is becoming increasing important to understand the financial impact of these providers on hospitals and health systems [10]. The development of an APP dashboard can provide a real-time snapshot to help health systems improve efficiency gaps and care team scalability. While there is no-one-size fits all solution to dashboard reporting, we have taken the opportunity to highlight steps our organization undertook in the creation of our novel APP dashboard report. By leveraging our in-house data analytic tools, we were able to create a highly visual report that categorizes APP productivity levels across wRVU performance targets using multiple volume-based metrics. Our success in the execution of an APP dashboard was facilitated by the selection of standardized financial and productivity metrics, which are generalizable and accessible at the individual, department, and enterprise level. Additionally, we used end-to-end data testing and validation procedures to ensure that our data was reliable and reflected accurately in the dashboard report. Lastly, benchmark patient satisfaction metrics will be included once APPs have been added to the contracted vendor’s surveying process.

## Data Availability

Data sharing is not applicable to this article as no datasets were generated or analyzed during the current study.

## Abbreviations

APP: Advanced Practice Providers
ETL: Extract, Transform, Load
VPN: Virtual Private Network
DUO: UCI Health’s software used for two factor authentication
PBG: UCI Health, SOM, Professional Billing Group
UCPath: The University of California’s payroll, benefits, human resources, and academic personnel system
KFS: Kuali Finance System software solution for core financial functions, including general ledger, procurement, budget, disbursing, and travel reimbursement
MGMA: Medical Group Management Association, https://www.mgma.com/
CPSC: Clinical Practice Solutions Center, AAMC and Vizient, https://www.clinicalpracticesolutionscenter.org/
Epic: UCI Heath’s Electronic Health Record (EHR) system, https://www.epic.com/
HAIS: UCI Health’s Information Services group
Tableau: Business Intelligence and analytics platform used by UCI School of Medicine for its reporting needs, https://www.tableau.com/
MS SQL: Microsoft SQL Server database platform, https://www.microsoft.com/en-us/sql-server?rtc=1
